# The Potential of Mesenchymal Stem Cells for the Treatment of Cytokine Storm due to COVID-19

**DOI:** 10.1155/2021/3178796

**Published:** 2021-11-26

**Authors:** Xun Li, Mengchao Yan, Jun Chen, Yang Luo

**Affiliations:** ^1^Key Laboratory of Biotherapy and Regenerative Medicine, Lanzhou, 730000 Gansu, China; ^2^The First Clinical Medical College, Lanzhou University, Lanzhou, 730000 Gansu, China; ^3^Medical Frontier Innovation Research Center, The First Hospital of Lanzhou University, Lanzhou, China; ^4^The Department of Neurology, The First Hospital of Lanzhou University, Lanzhou, 730000 Gansu, China

## Abstract

The outbreak of coronavirus disease 2019 (COVID-19) caused by severe acute respiratory syndrome coronavirus 2 (SARS-CoV-2) has seriously affected public health and social stability. The main route of the transmission is droplet transmission, where the oral cavity is the most important entry point to the body. Due to both the direct harmful effects of SARS-CoV-2 and disordered immune responses, some COVID-19 patients may progress to acute respiratory distress syndrome or even multiple organ failure. Genetic variants of SARS-CoV-2 have been emerging and circulating around the world. Currently, there is no internationally approved precise treatment for COVID-19. Mesenchymal stem cells (MSCs) can traffic and migrate towards the affected tissue, regulate both the innate and acquired immune systems, and participate in the process of healing. Here, we will discuss and investigate the mechanisms of immune disorder in COVID-19 and the therapeutic activity of MSCs, in particular human gingiva mesenchymal stem cells.

## 1. Introduction

The outbreak of a new coronavirus pneumonia (named COVID-19; previously known as 2019-nCoV) continues to cause a severe public health emergency worldwide. Epidemiological analysis shows that COVID-19 is an acute self-resolving disease, which can also be deadly, with an almost 2% case fatality rate [1–3]. Although the lung bears the brunt of the virus-induced damage, other parts of the body such as the liver, gastrointestinal tract, and heart are also affected [4–7]. Currently, there is no precise and effective treatment for COVID-19. Accumulating data from clinical case reports and basic research indicate that a hyperinflammatory response including cytokine storm possibly plays a role in the progression of COVID-19. Mesenchymal stem cells (MSCs), which are both immunosuppressive and immunomodulatory, are regarded as a promising therapeutic strategy in virus-induced hyperimmunoreactive disease, such as COVID-19. Gingival tissue-derived MSCs (GMSCs) have potent capacity for multidirectional differentiation and inflammatory modulation, making them an ideal subtype of MSCs for therapeutic use. In this review, we summarize the current understanding of the biology of the GMSC population and explore their potential therapeutic effects in virus-related diseases. We hypothesize that the administration of GMSCs could provide an innovative treatment for patients with COVID-19.

## 2. Clinical Characteristics of COVID-19 and Organ Involvement

**Figure 1:**
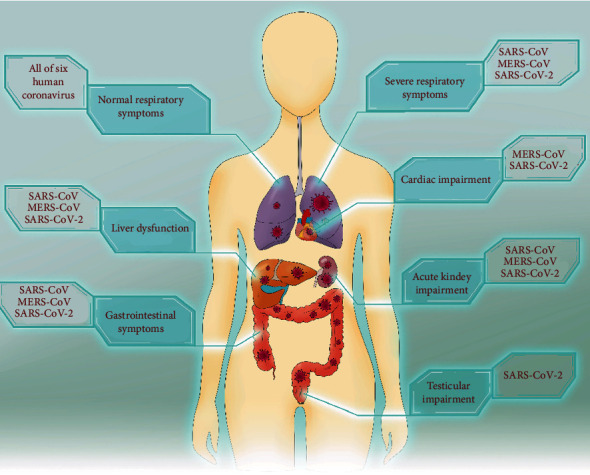
Systemic multisystem involvement of SARS-CoV-2 infection.

In general, all ages of the population are susceptible to SARS-CoV-2 infection; however, clinical manifestations differ with age. Notably, compared to young people and children, older men (>60 years old) with comorbidities are more likely to develop severe respiratory disease, requiring ventilation or monitoring in an intensive care unit (ICU) [8–10]. SARS-CoV-2 infection causes a series of systemic symptoms, such as fever, fatigue, dry cough, diarrhea, or even no symptoms at all [11–13]. Severe cases may involve organ dysfunction, including ARDS, acute cardiac injury, acute kidney injury, and even death, particularly when the patients have underlying diseases like hypertension, diabetes, and heart disease [12–15] (Figure 1). In addition, over 40% of COVID-19 patients have asymptomatic infection [16, 17].

Unfortunately, both symptomatic and asymptomatic patients can transmit the virus by the droplets and aerosols, in which the dental clinics could be one of the hardest hit areas for the infection [18, 19]. Blood tests show decreased leukocyte counts, prolonged prothrombin time, and elevated lactate dehydrogenase in most patients [20, 21]. Lung CT imaging indicates progressive infiltrate and diffuse gridding shadow in both lungs [20–22]. Currently available evidence indicates that SARS-CoV-2 likely emerged from a bat reservoir, although it remains unclear whether there are other animal species that acted as an intermediate host between bats and humans [23, 24].

Headache, dizziness, taste and smell dysfunctions, and impaired consciousness were the most frequently reported neurological symptoms in COVID-19 patients, each observed in more than five of the analyzed studies and with an overall frequency of over 4% of the populations studied. From the reported studies, headache was the most common symptom, which was more frequent in mild or moderate patients than severe ones. Rare symptoms such as acute cerebrovascular events and meningitis/encephalitis have also been observed in severely ill patients [6, 25, 26].

## 3. Coronaviral Structural Proteins and the Genome Structure of SARS-CoV-2

Coronaviruses belong to the virus family Coronaviridae, which are enveloped, nonsegmented, positive-sense, and single-stranded RNA virus genomes, infecting a variety of host species, including humans and several other vertebrates. As a novel betacoronavirus, SARS-CoV-2 shares 79% of its genome sequence identity with SARS-CoV and 50% with MERS-CoV; their genomes range approximately from 26 to 32 kilobases, making these viruses the largest known RNA viruses [27, 28]. They encode four major structural proteins: the spike protein (S), nucleocapsid protein (N), membrane protein (M), and the envelope protein (E), all of which are required to produce a structurally complete viral particle [29]. However, not all the proteins are required for forming a complete, infectious virion [30–32]. The role of each protein in the structure of the virus particle or involved in other aspects of the replication cycle mainly depends on the specific disease. In general, the S protein mediates attachment of the virus to the cell surface receptors and subsequently facilitates the viral entry process [32–34]. The N protein is the only protein that functions primarily to bind to the CoV RNA genome, usually making up the nucleocapsid [35, 36]. The M protein is the most abundant structural protein, which not only determines the shape of the viral envelope but is also regarded as the central organizer of CoV assembly [37, 38]. The E protein is the smallest of the major structural proteins and is abundantly expressed inside the infected cells during the replication cycle; however, only a small portion is assembled into the virion envelope [39]. Full-genome sequencing and phylogenic analysis demonstrated that SARS-CoV-2 is a novel clade probably from the betacoronaviruses that include bat-SARS-like (SL) ZC45, bat-SL ZXC21, SARS-CoV, and MERS-CoV [24]. The phylogenetic trees of the structural proteins are also clustered closely with those of the bat, civet, and human SARS coronaviruses [40]. Nevertheless, the external subdomain of the spike (S) receptor-binding domain of SARS-CoV-2 shares only 40% of its amino acid identity with other SARS-related coronaviruses [40]. Wrapp et al. dissected a 3.5 Å cryoelectron microscopy (cryo-EM) structure of the SARS-CoV-2 trimer in the prefusion conformation [41]. The predominant state of the trimer has one of the three receptor-binding domains (RBDs) rotated up in a receptor-accessible conformation. Finally, angiotensin-converting enzyme-2 (ACE2), the unequivocal functional receptor of SARS-CoV-2, encoded by a gene located on chromosome Xp22, plays a crucial role in the process of viral entry into the human cell. The spike (S) protein binds with ACE2 with higher affinity than (S) protein [42].

## 4. Immunopathogenesis of Coronavirus and SARS-CoV-2

Coronavirus's interaction with the host immune system plays an important role in determining the outcome of infection. The host's innate immune system spies on viral infections by activating pattern recognition receptors (PRRs) to recognize pathogen-associated molecular patterns (PAMPs). The IFN system is a crucial frontline defense against viral infections and spread. IFN production-related PRRs mainly include TLRs, RLRs, and NLRs [43]. Type I IFNs (particularly IFN-*α* and IFN-*β*) activate the downstream JAK-STAT signal pathway, promoting the expression of IFN-stimulated genes (ISGs), subsequently mediating antiviral effects by directly inhibiting coronavirus replication and indirectly modulating the host immune response [44, 45]. Rapid coronavirus replication reaching high titers and associated with enhanced inflammatory responses, such as an unregulated production of IFNs, is believed to result in cytokine storm [46]. Virus-associated cytokine storm is characterized by an immunogenic cascade reaction. After infection, the highly pathogenic hCoVs may lead to delayed IFN production *via* multiple structural and nonstructural proteins [47]. Unrestrained virus replication and more viral PAMPs may result in excessive release of more proinflammatory cytokines, recruitment of a large number of inflammatory cells, and an aberrant cascade of inflammatory responses [48]. Research has shown that SARS-CoV-2 can promote autophagy, which plays a crucial role in suppressing the type I interferon response [49]. The hypercytokinemia and systemic immunopathology lead to a progressive immune-associated injury resulting in severe pneumonia [50]. In patients with severe illness, high levels of proinflammatory cytokines (IFNs, IL-1, IL-6, IL-12, and TGF-*β*) and chemokines (CCL2, CXCL10, CXCL9, and IL-8) were found in serum [51–53].

Recent studies have suggested that the pathophysiology of SARS-CoV-2 infection is due not only to the damage caused by the virus itself but also to the host response. It is certain that uncontrolled inflammation, also referred to as cytokine storm, contributes to disease severity in COVID-19 [54]. Huang et al. analyzed the immunological features of peripheral blood from 40 confirmed patients. They reported that about 25% patients had leucopenia and approximately 63% had lymphopenia [13]. Liu et al. also observed a dozen patients and found that the more severe the disease, the higher the prothrombin time and D-dimer levels [20]. In addition, aspartate aminotransferase and hypersensitive troponin I (hs-cTnI) were mildly increased compared to the levels seen in general pneumonia. An uncontrolled systemic inflammatory response results from the release of large amounts of proinflammatory cytokines (IFN*α*, IFN*γ*, IL-1*β*, IL-6, IL-12, IL-17, IL-18, IL-33, TNF-*α*, TGF*β*, etc.) and chemokines (CCL2, CCL3, CCL5, CXCL8, CXCL9, CXCL10, etc.) by immune cells [55]. Interestingly, the plasma concentrations of IL-2, IL-7, IL-10, G-CSF, IP10, MCP-1, and TNF*α* were significantly higher in ICU patients than non-ICU patients. This provides preliminary evidence that the extent of the hypercytokinemia may predict the clinical consequences. Qin et al. observed abnormal changes in the adaptive immune response in COVID-19 cases. The levels of both suppressor T cells (CD3^+^CD8^+^) and T helper cells (CD3^+^ CD4^+^) were below normal. Meanwhile, the percentage of naïve T cells (CD3^+^CD4^+^CD45RA^+^) was increased, and that of memory T cells (CD3^+^CD4^+^CD45RO^+^) was decreased in severe cases, indicating the severity of immune system impairment [55]. Compared to mildly ill patients, most severe cases of COVID-19 have lower percentages of monocytes, eosinophils, and basophils [56]. Using RNA sequencing combined with single-cell proteomics, one research group determined that elevated frequency of HLA-DR^hi^CD11c^hi^ inflammatory monocytes with an IFN-stimulated gene signature was found in mild COVID-19, whereas severe COVID-19 was characterized by the occurrence of neutrophil precursors, as evidence of emergency myelopoiesis, dysfunctional mature neutrophils, and HLA-DR^lo^ monocytes [57].

## 5. Mesenchymal Stem Cells

Up to now, more than 150 clinical trials have been launched to test coronavirus treatments all over the world (https://www.who.int/ictrp/zh/). Although there are several vaccines that are effective in preventing the spread of COVID-19, no drugs are available to specifically treat COVID-19 patients [58]. Previous studies reported on the safety and applicability of mesenchymal stem/stromal cells (MSCs) to ameliorate pulmonary inflammation in acute respiratory distress syndrome [59]. In light of this, MSC-based immunomodulation treatment has been proposed as a powerful therapeutic approach against COVID-19.

### 5.1. Characteristic of MSCs

**Figure 2:**
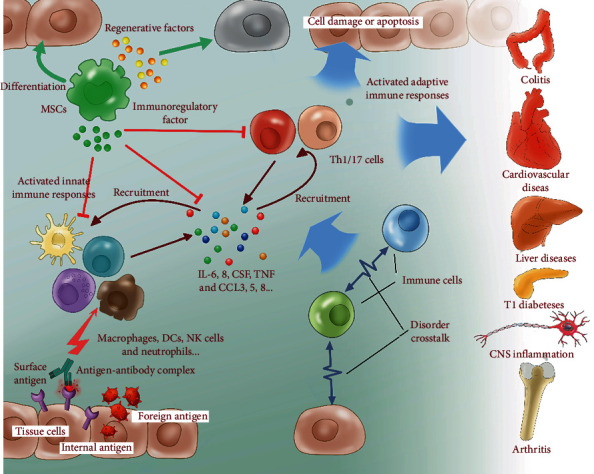
MSC therapy produces meaningful therapeutic outcomes in the treatment of pulmonary, cardiovascular, neurological, liver, kidney, arthritic, and CNS inflammatory diseases.

Stem cells can be split into two major groups: embryonic and nonembryonic. Among nonembryonic stem cells, MSCs represent an intensively investigated population with unique biological properties [60]. Similar subsets of multipotent MSCs have been identified in dental pulp, skin, umbilical cord blood, and adipose tissue [61]. Cs usually express specific genes for embryonic stem cells, such as octamer-4 (Oct-4) and stage-specific embryonic Ag 4 (SSEA-4), and share a similar expression profile of cell surface molecules, such as CD105, CD73, CD90, CD146, and CD29, but typically lack hematopoietic stem cell markers, such as CD34 and CD45 [62–64]. All of these MSC subsets have the capacity for self-proliferation and multidifferentiation. In addition, they also display chemotactic, anti-inflammatory, and immunomodulatory properties, similar to immune regulatory cells, in response to tissue insult and inflammation via production of anti-inflammatory cytokines and antiapoptotic molecules [65]. Indeed, immune regulatory cells have potent functional capacity to suppress immune response and control inflammatory diseases [66]. MSCs' unique characteristics have led to the suggestion that MSC-based therapies provide a potential approach to controlling inflammation in the repair or regeneration of a variety of damaged tissues and organs (Figure 2).

### 5.2. The Paracrine System, Homing Effects, and Immunomodulation

**Figure 3:**
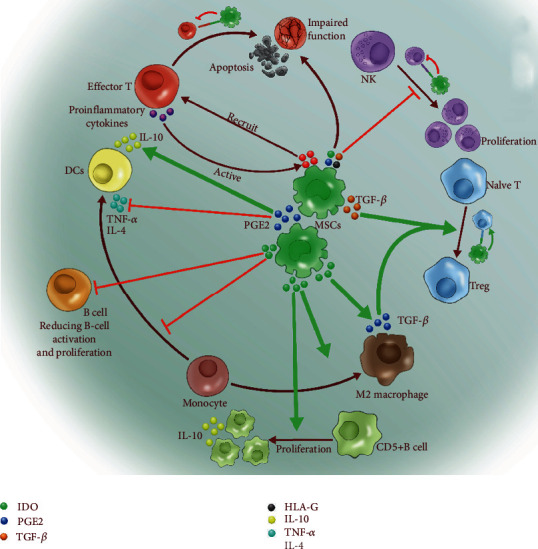
MSCs can alter the behavior of both adaptive and innate immune cells, regulating the condition of a variety of pathological microenvironments.

A growing body of evidence has demonstrated that MSCs have the potential to secrete a wide variety of cytokines, chemokines, and growth factors, which exert profound effects when they interact with the microenvironments mediating the tissue function [67, 68]. The MSC secretome identified which released factors are at high levels, such as proteins involved in immune system signaling (i.e., IL-6, IL-8, MCP-1, and TGF-*β*), extracellular matrix remodelers (i.e., TIMP-2, fibronectin, periostin, collagen, and decorin), and growth factors and their regulators (i.e., VEGF, GM-CSF, BMP-2, and IGFBPs) [69–71]. Moreover, MSC-conditioned media also act as a chemoattractant for recruiting macrophages and endothelial cells into wound tissue to enhance the healing process or decrease the cardiac infarct size [66, 72]. The homing mechanism of MSCs involves several cell trafficking-related molecules such as chemokines, adhesion molecules, and matrix metalloproteinase [73]. Among them, CCR-2/3, CXCR-4, VLA-4, and CXCR-9 are the most important signalers [74, 75]. In order to reach the injured tissue, MSCs exhibit transendothelial migration ability, in adhering to vascular endothelial cells and crossing the endothelial barrier. In this process, several MMPs have proven to increase the invasiveness of MSCs [76]. MSCs exert their immunomodulatory function mostly dependent on cell-to-cell contact and/or the release of soluble immunosuppressive factors [77] (Figure 3). A series of studies have demonstrated that MSCs interact with a wide range of immune cells and display an ability to suppress the excessive response of T and B lymphocytes, dendritic cells, macrophages, mast cells, and natural killer cells, as well as promote the expansion of regulatory T cells [78–81]. For the crosstalk with Treg cells, short-lived MSCs can act as catalysts in induction and expansion of long-lasting antigen-specific Treg cells to continue the immunosuppressive capacity [82, 83]. In cytoimmunotherapy, MSCs could become the gold standard for the treatment of organ damage associated with intense inflammatory activity (e.g., rheumatoid arthritis, kidney failure, heart injury, GVHD, systemic lupus erythematosus, and multiple sclerosis) [84].

### 5.3. MSCs from Different Sources Have Different Functions against Virus Infection

Source-related features of MSCs directly contribute to the diversity of opinions regarding the mechanisms of MSC-mediated immunomodulation. In terms of current clinical applications, the main sources of MSCs are bone marrow (BM), adipose tissue (AT), and umbilical cord (UC) [85]. BM-MSC separation is painful for the patient and is accompanied by a risk of infection. Pittenger et al. demonstrated that only 0.001 to 0.01% of the cells are real mesenchymal stem cells when extracted by density gradient centrifugation. Functionally, BM-MSCs possess a longer duplication period and reach senescence earlier. However, several basic and clinical studies showed that lower immunomodulatory activity of BM-MSCs in an inflammatory environment *in vitro* and poor therapeutic effects were observed in a real-world study [85, 86].

AT-MSCs have been shown to have higher proliferation capacities than BM-MSCs, with population doubling times of 45.2 h for AT-MSCs compared to 61.2 h for BM-MSCs, illustrated by Peng et al. [87]. AD-MSCs also avoid the ethical problems of BM-MSCs. Multiple clinical trials have proven that AD-MSCs can treat arthritis, diabetes, and heart failure and achieve good outcomes [88, 89]. It should, however, be noted that the heterogeneity of AT-MSCs varies with different regions of the body, posing a challenge for clinical application [90]. In comparison, umbilical cord-derived MSCs (UC-MSCs) are more primitive and immunosuppressive than their adult counterparts. Nevertheless, in terms of these three products, there are still many questions regarding the clinical application of MSCs that need to be answered, and further studies are warranted, such as the effect of donor selection, long-term therapeutic effects, product consistency, and potential tumorigenicity [91].

### 5.4. Potential Mechanism of MSCs against SARS-CoV-2

**Figure 4:**
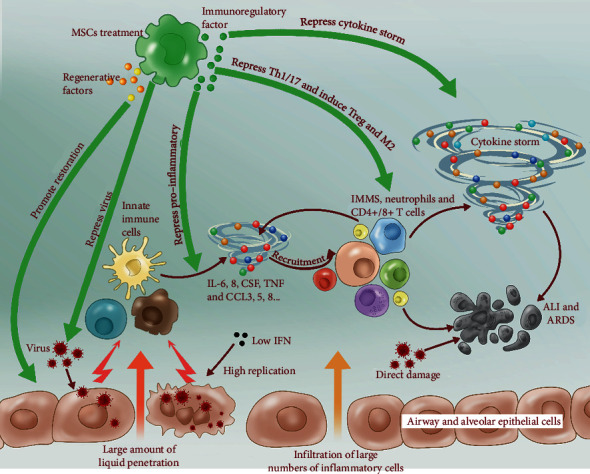
Schematic of the potential mechanism of MSC action and host immune system responses during SARS-CoV-2 infection.

Coronaviruses, such as SARS, MERS, and even SARS-CoV-2, continuously undergo mutations resulting in the generation of new viral strains that can become resistant to antiviral drugs [92, 93]. MSC therapy has several mechanisms of action making it unlikely that the virus could develop resistance to this treatment (Figure 4). MSC administration had beneficial effects on ARDS in animal models [94, 95]. MSCs were shown not only to repress the activities of influenza viruses but also to directly inhibit replication and virus-induced apoptosis in lung epithelial cells [96]. Furthermore, the production of the proinflammatory cytokine TNF*α* and the chemokine CXCL10 was significantly decreased after MSC administration, accompanied by an increased production of IL-10 [96], a potent anti-inflammatory cytokine za A (H5N1) virus also causes acute lung injury, and two groups reported that hUC-MSCs and BM-MSCs were effective in restoring impaired alveolar fluid clearance and protein permeability of A(H5N1)-infected human alveolar epithelial cells [97, 98].

Clinical trials are ongoing across the world to evaluate the efficacy of cell-based therapy against COVID-19. A case study was reported of an acute SARS-CoV-2 infected female patient with poor oxygenation, who received hUC-MSCs by intravenous infusion. After three weeks of dynamic observation, the results of blood tests and CT images provided evidence of an extremely good prognosis [5, 99]. In another study reported recently in China, patients with severe COVID-19 were randomly divided into 2 groups: the standard treatment group and the standard treatment plus hUC-MSC infusion group (single dose of 10^6^ UC-MSC/kg). The results showed that the MSC-treated group had greater clinical improvement than the control group, accompanied by lower CRP and IL-6 levels in peripheral blood and faster lung inflammation absorption [100]. Also, the gene expression profile showed that MSCs were ACE2 negative, which means that transplanted MSCs did not differentiate and remained free of virus [99, 100]. Results from the phase I-II and multicenter study (ChiCTR2000029990) showed that overactivated immune cells (CXCR3^+^CD4^+^T cells, CXCR3^+^CD8^+^ T cells, and CXCR3^+^NK cells) and serum TNF-*α* and IL-6 levels were significantly decreased, while anti-inflammatory IL-10 levels were increased in the MSC treatment group [100, 101]. Mechanically, human bone marrow-MSCs were negative for ACE2 and TMPRSS2 genes, suggesting that human BM-MSCs may be free from SARS-CoV-2 infection and its immunomodulatory properties might be maintained under the virus microenvironment [102]. Meanwhile, MSCs possess the capacity for tissue regeneration and cytokine storm suppression in treating ARDS, which were also applied to fight against COVID-19.

## 6. Human Gingiva Mesenchymal Stem Cells

### 6.1. Characteristics and Functions of GMSCs

The human gingiva is a tissue that not only is easily obtained from the oral cavity but also can be used as a discarded biological sample. Human gingiva MSCs (GMSCs) are capable of eliciting a potent inhibitory effect on peripheral blood lymphocyte proliferation and cytokine production [103]. Most importantly, GMSCs express a wide panel of immunosuppressive factors including IL-10, IDO, inducible NO synthase (iNOS), and cyclooxygenase 2 (COX-2), in response to the inflammatory milieu [104].

GMSC transplantation was shown to effectively alleviate the arthritis symptoms of mice in collagen-induced arthritis (CIA) and ameliorate immune-mediated bone marrow failure of aplastic anemia (AA) [105]. Additionally, our group found that GMSCs can generate adenosine *via* extracellular enzymes CD39 and CD73, which can inhibit the differentiation of osteoclastogenesis and promote osteoblasts *via* the Wnt/*β*-Catenin pathway [106]. In a diabetes model, we confirmed that GMSCs even enhanced their suppressive function in inflammation and that the microRNA-21a-5p/PDCD4 axis regulates their functional activities [107, 108]. Studies using several mouse models revealed that GMSC transplantation can prevent experimental colitis and alleviate the oral cavity mucosal inflammation induced by chemotherapy [109, 110]. In a preclinical study, we demonstrated that the administration of GMSCs is very safe. In addition to possessing stem cell-like properties and immunomodulatory functions, GMSCs also have the following special biological characteristics, compared to other MSCs: (1) they are easy to isolate and culture and proliferate faster than BM-MSCs; (2) they have no tumorigenesis and maintain a stable and uniform phenotype after long-term cultivation; and (3) whether from autoimmune patients or healthy volunteers, their cellular properties and physiological functions remain unchanged, which implies that the autologous GMSCs can be applied to treat relevant diseases [111, 112].

### 6.2. GMSCs against SARS-CoV-2

From the autopsy results of a SARS-CoV-2-infected pneumonia patient, histological examination showed bilateral diffuse alveolar damage with cellular fibromyxoid exudates and interstitial mononuclear inflammatory infiltrates in both lungs, dominated by lymphocytes [2]. The main manifestation was an excessive inflammatory response. Although peripheral CD4^+^ and CD8^+^ T cells were substantially reduced, they were overactivated, as evidenced by the high proportions of HLA-DR and CD38, accompanied by increased concentration of CCR4^+^ CCR6^+^ Th17 cells. Besides, CD8^+^ T cells were found to harbor high concentrations of cytotoxic granules, of which a few were perforin positive and some were granulysin positive. From this, we can speculate that the redistribution of lymphocytes in the infected body may contribute to peripheral blood lymphocytopenia and increased lymphocyte infiltration in lung tissue. In other words, the immune system excessively mobilizes lymphocytes to migrate to the pneumonic lungs, or virus-infected lung tissue produces some chemotactic factors that attract the lymphocytes' migration. A controversial question currently is whether the acute liver injury seen in some COVID-19 patients is SARS-CoV-2-caused or drug-induced. It is more likely that it is due to the cytokine storm, a virus-triggered immune overreaction. Ahmadi et al. performed an analysis of the CD39 and CD73 expression pattern on CD4^+^ T, CD8^+^ T, and natural killer T cells of COVID-19 using a flow cytometry panel [42]. The results were a correlation between the absence of CD73 from CD8^+^ T cells and NKT and increased ability to secrete granzyme B, perforin, TNF-*α*, and IFN-*γ* regardless of the disease status. Another study also confirmed that SARS-CoV-2 can exhaust CD8 T lymphocytes with elevated CD39 and TIM-3 exhaustion markers. Studies from our group showed that human GMSCs highly expressed CD39/CD73, contributing to the therapeutic effect on several autoimmune inflammatory diseases. Because of the advantage, GMSC may be more effective on treating COVID-19.

## 7. Conclusions

Although COVID-19 therapies have targeted various pathogenic mechanisms, there are no established treatments currently. The therapeutic potential of GMSC-based cell therapy against the SARS-CoV-2-related diseases is explained here. Multiple ongoing trials are now testing MSCs in patients with severe COVID-19, and pilot uncontrolled trials have reported promising results. However, the efficacy and side effects of MSCs therapy should be confirmed in larger trials. Human gingiva MSCs have great potential, and their clinical application needs to be carefully designed.

## Data Availability

The data and materials used during the current review are all available in this review.

## Abbreviations

ACE2: Angiotensin-converting enzyme 2
ARDS: Acute respiratory distress syndrome
CRP: C-reactive protein
COVID-19: Coronavirus disease 2019
CT: Computed tomography
IFN-*γ*: Interferon gamma
MSCs: Mesenchymal stem cells
RBD: Receptor-binding domain
SARS-CoV-2: Severe acute respiratory syndrome coronavirus 2
S protein: Spike protein.

## References

[1] Battegay M., Kuehl R., Tschudin-Sutter S., Hirsch H. H., Widmer A. F., Neher R. A. (2020). 2019-novel coronavirus (2019-nCoV): estimating the case fatality rate - a word of caution. *Swiss Medical Weekly*.

[2] Xu Z., Shi L., Wang Y. (2020). Pathological findings of COVID-19 associated with acute respiratory distress syndrome. *The Lancet Respiratory Medicine*.

[3] Salama C., Han J., Yau L. (2021). Tocilizumab in patients hospitalized with Covid-19 pneumonia. *New England Journal of Medicine*.

[4] Liu K., Fang Y. Y., Deng Y. (2020). Clinical characteristics of novel coronavirus cases in tertiary hospitals in Hubei Province. *Chinese medical journal*.

[5] Shu L., Niu C., Li R. (2020). Treatment of severe COVID-19 with human umbilical cord mesenchymal stem cells. *Stem Cell Research & Therapy*.

[6] Iadecola C., Anrather J., Kamel H. (2020). Effects of COVID-19 on the nervous system. *Cell*.

[7] Dashraath P., Nielsen-Saines K., Madhi S. A., Baud D. (2020). COVID-19 vaccines and neglected pregnancy. *Lancet*.

[8] Hu B., Guo H., Zhou P., Shi Z. L. (2021). Characteristics of SARS-CoV-2 and COVID-19. *Nature Reviews Microbiology*.

[9] Yuan L., Zhu H., Zhou M. (2021). Gender associates with both susceptibility to infection and pathogenesis of SARS-CoV-2 in Syrian hamster. *Signal Transduction and Targeted Therapy*.

[10] Jin Y. H., Zhan Q. Y., Peng Z. Y. (2020). Chemoprophylaxis, diagnosis, treatments, and discharge management of COVID-19: an evidence-based clinical practice guideline (updated version). *Military Medical Research*.

[11] Cervino G., Oteri G. (2020). COVID-19 pandemic and telephone triage before attending medical office: problem or opportunity?. *Medicina (Kaunas, Lithuania)*.

[12] Wang D., Hu B., Hu C. (2020). Clinical characteristics of 138 hospitalized patients with 2019 novel coronavirus-infected pneumonia in Wuhan, China. *JAMA*.

[13] Wang D., Hu B., Hu C. (2020). Clinical features of patients infected with 2019 novel coronavirus in Wuhan, China. *Lancet*.

[14] Gandhi R. T., Lynch J. B., Del Rio C. (2020). Mild or moderate Covid-19. *New England Journal of Medicine*.

[15] Adedokun K. A. (2021). Early stage nonclinical pulmonary disorder in COVID-19 may present asymptomatic and fuel the contagion. *Military Medical Research*.

[16] Sui Z., Dai X., Lu Q. (2021). Viral dynamics and antibody responses in people with asymptomatic SARS-CoV-2 infection. *Signal Transduction and Targeted Therapy*.

[17] Fiorillo L., Cervino G., Matarese M. (2020). COVID-19 surface persistence: a recent data summary and its importance for medical and dental settings. *International Journal of Environmental Research and Public Health*.

[18] Fiorillo L., Meto A., Cicciù F., De Stefano R. (2021). An eventual Sars-CoV-2 infection prevention protocol in the medical setting and dental office. *International Journal of Environmental Research and Public Health*.

[19] Cicciù M., Fiorillo L., Laino L. (2021). Oral signs and symptoms of COVID-19 affected patients: dental practice as prevention method. *Minerva Dental and Oral Science*.

[20] Liu Y., Yang Y., Zhang C. (2020). Clinical and biochemical indexes from 2019-nCoV infected patients linked to viral loads and lung injury. *Science China. Life Sciences*.

[21] Zhang J. J., Dong X., Cao Y. Y. (2020). Clinical characteristics of 140 patients infected with SARS-CoV-2 in Wuhan, China. *Allergy*.

[22] Sanli D. E. T., Yildirim D., Sanli A. N. (2021). A practical approach to imaging characteristics and standardized reporting of COVID-19: a radiologic review. *Military Medical Research*.

[23] Chen L., Liu W., Zhang Q. (2020). RNA based mNGS approach identifies a novel human coronavirus from two individual pneumonia cases in 2019 Wuhan outbreak. *Emerging microbes and infections*.

[24] Lu R., Zhao X., Li J. (2020). Genomic characterisation and epidemiology of 2019 novel coronavirus: implications for virus origins and receptor binding. *Lancet*.

[25] Chen X., Laurent S., Onur O. A. (2021). A systematic review of neurological symptoms and complications of COVID-19. *Journal of Neurology*.

[26] Zhang L. B., Pang R. R., Qiao Q. H. (2020). Successful recovery of COVID-19-associated recurrent diarrhea and gastrointestinal hemorrhage using convalescent plasma. *Military Medical Research*.

[27] Weiss P., Murdoch D. R. (2020). Clinical course and mortality risk of severe COVID-19. *Lancet*.

[28] Muñoz-Fontela C., Dowling W. E., Funnell S. G. P. (2020). Animal models for COVID-19. *Nature*.

[29] Masters P. S. (2006). The molecular biology of coronaviruses. *Advances in Virus Research*.

[30] Kuo L., Masters P. S. (2003). The small envelope protein E is not essential for murine coronavirus replication. *Journal of Virology*.

[31] Ortego J., Ceriani J. E., Patino C., Plana J., Enjuanes L. (2007). Absence of E protein arrests transmissible gastroenteritis coronavirus maturation in the secretory pathway. *Virology*.

[32] Siu Y. L., Teoh K. T., Lo J. (2008). The M, E, and N structural proteins of the severe acute respiratory syndrome coronavirus are required for efficient assembly, trafficking, and release of virus-like particles. *Journal of Virology*.

[33] Song H. C., Seo M. Y., Stadler K. (2004). Synthesis and characterization of a native, oligomeric form of recombinant severe acute respiratory syndrome coronavirus spike glycoprotein. *Journal of Virology*.

[34] Kirchdoerfer R. N., Cottrell C. A., Wang N. (2016). Pre-fusion structure of a human coronavirus spike protein. *Nature*.

[35] de Haan C. A., Rottier P. J. (2005). Molecular interactions in the assembly of coronaviruses. *Advances in Virus Research*.

[36] McBride R., van Zyl M., Fielding B. C. (2014). The coronavirus nucleocapsid is a multifunctional protein. *Viruses*.

[37] Neuman B. W., Kiss G., Kunding A. H. (2011). A structural analysis of M protein in coronavirus assembly and morphology. *Journal of Structural Biology*.

[38] Lim K. P., Liu D. X. (2001). The Missing Link in Coronavirus Assembly:. *Journal of Biological Chemistry*.

[39] Venkatagopalan P., Daskalova S. M., Lopez L. A., Dolezal K. A., Hogue B. G. (2015). Coronavirus envelope (E) protein remains at the site of assembly. *Virology*.

[40] Chan J. F., Kok K. H., Zhu Z. (2020). Genomic characterization of the 2019 novel human-pathogenic coronavirus isolated from a patient with atypical pneumonia after visiting Wuhan. *Emerging Microbes & Infections*.

[41] Wrapp D., Wang N., Corbett K. S. (2020). Cryo-EM structure of the 2019-nCoV spike in the prefusion conformation. *Science*.

[42] Bourgonje A. R., Abdulle A. E., Timens W. (2020). Angiotensin‐converting enzyme 2 (ACE2), SARS‐CoV‐2 and the pathophysiology of coronavirus disease 2019 (COVID‐19). *The Journal of Pathology*.

[43] Baccala R., Gonzalez-Quintial R., Lawson B. R. (2009). Sensors of the innate immune system: their mode of action. *Nature Reviews Rheumatology*.

[44] Ma D. Y., Suthar M. S. (2015). Mechanisms of innate immune evasion in re-emerging RNA viruses. *Current Opinion in Virology*.

[45] Nelemans T., Kikkert M. (2019). Viral innate immune evasion and the pathogenesis of emerging RNA virus infections. *Viruses*.

[46] Channappanavar R., Perlman S. (2017). Pathogenic human coronavirus infections: causes and consequences of cytokine storm and immunopathology. *Seminars in Immunopathology*.

[47] Siu K.-L., Kok K.-H., Ng M.-H. J. (2009). Severe Acute Respiratory Syndrome Coronavirus M Protein Inhibits Type I Interferon Production by Impeding the Formation of TRAF3∗TANK∗TBK1/IKK*ϵ* Complex∗. *Journal of Biological Chemistry*.

[48] Channappanavar R., Fehr A. R., Vijay R. (2016). Dysregulated type I interferon and inflammatory monocyte-macrophage responses cause lethal pneumonia in SARS-CoV-infected mice. *Cell Host Microbe*.

[49] Hui X., Zhang L., Cao L. (2021). SARS-CoV-2 promote autophagy to suppress type I interferon response. *Signal Transduction and Targeted Therapy*.

[50] Huang K. J., Su I. J., Theron M. (2005). An interferon-?-related cytokine storm in SARS patients. *Journal of Medical Virology*.

[51] Wong C. K., Lam C. W. K., Wu A. K. L. (2004). Plasma inflammatory cytokines and chemokines in severe acute respiratory syndrome. *Clinical & Experimental Immunology*.

[52] Zhang Y., Li J., Zhan Y. (2004). Analysis of serum cytokines in patients with severe acute respiratory syndrome. *Infection and Immunity*.

[53] Kim E. S., Choe P. G., Park W. B. (2016). Clinical progression and cytokine profiles of Middle East respiratory syndrome coronavirus infection. *Journal of Korean Medical Science*.

[54] Zhang J. Y., Wang X. M., Xing X. (2020). Single-cell landscape of immunological responses in patients with COVID-19. *Nature Immunology*.

[55] Muyayalo K. P., Huang D. H., Zhao S. J., Xie T., Mor G., Liao A. H. (2020). COVID-19 and Treg/Th17 imbalance: potential relationship to pregnancy outcomes. *American Journal of Reproductive Immunology*.

[56] Zhang B., Zhou X., Zhu C. (2020). Immune phenotyping based on the neutrophil-to-lymphocyte ratio and IgG level predicts disease severity and outcome for patients with COVID-19. *Frontiers in Molecular Biosciences*.

[57] Schulte-Schrepping J., Reusch N., Paclik D. (2020). Severe COVID-19 is marked by a dysregulated myeloid cell compartment. *Cell*.

[58] Yang R., Deng Y., Huang B. (2021). A core-shell structured COVID-19 mRNA vaccine with favorable biodistribution pattern and promising immunity. *Signal Transduction and Targeted Therapy*.

[59] Fujita Y., Kadota T., Araya J., Ochiya T., Kuwano K. (2018). Clinical application of mesenchymal stem cell-derived extracellular vesicle-based therapeutics for inflammatory lung diseases. *Journal of Clinical Medicine*.

[60] He Q., Ye Z., Zhou Y., Tan W. S. (2018). Comparative study of mesenchymal stem cells from rat bone marrow and adipose tissue. *Turkish Journal of Biology*.

[61] Mazini L., Rochette L., Admou B., Amal S., Malka G. (2020). Hopes and limits of adipose-derived stem cells (ADSCs) and mesenchymal stem cells (MSCs) in wound healing. *International Journal of Molecular Sciences*.

[62] Dominici M., le Blanc K., Mueller I. (2006). Minimal criteria for defining multipotent mesenchymal stromal cells. The International Society for Cellular Therapy position statement. *Cytotherapy*.

[63] Samsonraj R. M., Raghunath M., Nurcombe V., Hui J. H., van Wijnen A. J., Cool S. M. (2017). Concise review: multifaceted characterization of human mesenchymal stem cells for use in regenerative medicine. *STEM CELLS Translational Medicine*.

[64] Lv F. J., Tuan R. S., Cheung K. M., Leung V. Y. (2014). Concise review: the surface markers and identity of human mesenchymal stem cells. *Stem Cells*.

[65] Shi Y., Wang Y., Li Q. (2018). Immunoregulatory mechanisms of mesenchymal stem and stromal cells in inflammatory diseases. *Nature Reviews Nephrology*.

[66] Chen L., Xu Y., Zhao J. (2014). Conditioned medium from hypoxic bone marrow-derived mesenchymal stem cells enhances wound healing in mice. *PLoS One*.

[67] Fang S. B., Zhang H. Y., Wang C. (2020). Small extracellular vesicles derived from human mesenchymal stromal cells prevent group 2 innate lymphoid cell-dominant allergic airway inflammation through delivery of miR-146a-5p. *Journal of Extracellular Vesicles*.

[68] Su W., Wan Q., Huang J. (2015). Culture medium from TNF-*α*-stimulated mesenchymal stem cells attenuates allergic conjunctivitis through multiple antiallergic mechanisms. *Journal of Allergy and Clinical Immunology*.

[69] Wei X., Yang X., Han Z. P., Qu F. F., Shao L., Shi Y. F. (2013). Mesenchymal stem cells: a new trend for cell therapy. *Acta Pharmacologica Sinica*.

[70] Prockop D. J., Youn Oh J. (2012). Mesenchymal stem/stromal cells (MSCs): role as guardians of inflammation. *Molecular Therapy*.

[71] Park W. S., Ahn S. Y., Sung S. I., Ahn J. Y., Chang Y. S. (2018). Strategies to enhance paracrine potency of transplanted mesenchymal stem cells in intractable neonatal disorders. *Pediatric Research*.

[72] Timmers L., Lim S. K., Arslan F. (2008). Reduction of myocardial infarct size by human mesenchymal stem cell conditioned medium. *Stem Cell Research*.

[73] Steingen C., Brenig F., Baumgartner L., Schmidt J., Schmidt A., Bloch W. (2008). Characterization of key mechanisms in transmigration and invasion of mesenchymal stem cells. *Journal of Molecular and Cellular Cardiology*.

[74] Jimenez-Puerta G. J., Marchal J. A., Lopez-Ruiz E., Galvez-Martin P. (2020). Role of mesenchymal stromal cells as therapeutic agents: potential mechanisms of action and implications in their clinical use. *Journal of Clinical Medicine*.

[75] Nitzsche F., Müller C., Lukomska B., Jolkkonen J., Deten A., Boltze J. (2017). Concise review: MSC adhesion cascade-insights into homing and transendothelial migration. *Stem Cells*.

[76] Ries C., Egea V., Karow M., Kolb H., Jochum M., Neth P. (2007). MMP-2, MT1-MMP, and TIMP-2 are essential for the invasive capacity of human mesenchymal stem cells: differential regulation by inflammatory cytokines. *Blood*.

[77] Gao F., Chiu S. M., Motan D. A. (2016). Mesenchymal stem cells and immunomodulation: current status and future prospects. *Cell Death & Disease*.

[78] Yanez R., Lamana M. L., Garcia-Castro J., Colmenero I., Ramirez M., Bueren J. A. (2006). Adipose tissue-derived mesenchymal stem cells have in vivo immunosuppressive properties applicable for the control of the graft-versus-host disease. *Stem Cells*.

[79] Glennie S., Soeiro I., Dyson P. J., Lam E. W., Dazzi F. (2005). Bone marrow mesenchymal stem cells induce division arrest anergy of activated T cells. *Blood*.

[80] Corcione A., Benvenuto F., Ferretti E. (2006). Human mesenchymal stem cells modulate B-cell functions. *Blood*.

[81] Barbaro N. R., Foss J. D., Kryshtal D. O. (2017). Dendritic cell amiloride-sensitive channels mediate sodium-induced inflammation and hypertension. *Cell Reports*.

[82] Selmani Z., Naji A., Zidi I. (2008). Human leukocyte antigen-G5 secretion by human mesenchymal stem cells is required to suppress T lymphocyte and natural killer function and to induce CD4+CD25highFOXP3+ regulatory T cells. *Stem Cells*.

[83] English K., Ryan J. M., Tobin L., Murphy M. J., Barry F. P., Mahon B. P. (2009). Cell contact, prostaglandin E_2_ and transforming growth factor beta 1 play non-redundant roles in human mesenchymal stem cell induction of CD4^+^CD25^High^forkhead box P3+regulatory T cells. *Clinical & Experimental Immunology*.

[84] Wang L. T., Ting C. H., Yen M. L. (2016). Human mesenchymal stem cells (MSCs) for treatment towards immune- and inflammation-mediated diseases: review of current clinical trials. *Journal of Biomedical Science*.

[85] Berebichez-Fridman R., Montero-Olvera P. R. (2018). Sources and clinical applications of mesenchymal stem cells: state-of-the-art review. *Sultan Qaboos University Medical Journal [SQUMJ]*.

[86] Berthelot J. M., Le Goff B., Maugars Y. (2019). Bone marrow mesenchymal stem cells in rheumatoid arthritis, spondyloarthritis, and ankylosing spondylitis: problems rather than solutions?. *Arthritis Research & Therapy*.

[87] Peng L., Jia Z., Yin X. (2008). Comparative analysis of mesenchymal stem cells from bone marrow, cartilage, and adipose tissue. *Stem Cells and Development*.

[88] Bartolucci J., Verdugo F. J., González P. L. (2017). Safety and efficacy of the intravenous infusion of umbilical cord mesenchymal stem cells in patients with heart failure: a phase 1/2 randomized controlled trial (RIMECARD trial [Randomized Clinical Trial of Intravenous Infusion Umbilical Cord Mesenchymal Stem Cells on Cardiopathy]). *Circulation Research*.

[89] Chahal J., Gómez-Aristizábal A., Shestopaloff K. (2019). Bone marrow mesenchymal stromal Cells in patients with osteoarthritis results in overall improvement in pain and symptoms and reduces synovial inflammation. *STEM CELLS Translational Medicine*.

[90] Hass R., Kasper C., Böhm S., Jacobs R. (2011). Different populations and sources of human mesenchymal stem cells (MSC): a comparison of adult and neonatal tissue-derived MSC. *Cell Communication and Signaling*.

[91] Wu K. H., Wu H. P., Chan C. K., Hwang S. M., Peng C. T., Chao Y. H. (2013). The role of mesenchymal stem cells in hematopoietic stem cell transplantation: from bench to bedsides. *Cell Transplantation*.

[92] Phan T. (2020). Genetic diversity and evolution of SARS-CoV-2. *Infection, Genetics and Evolution*.

[93] Kleine-Weber H., Elzayat M. T., Wang L. (2019). Mutations in the spike protein of Middle East respiratory syndrome coronavirus transmitted in Korea increase resistance to antibody-mediated neutralization. *Journal of Virology*.

[94] Wang H. Z., Yang C., Zhang B. Y., Li N., Han Z., Chen F. (2019). Influence of mesenchymal stem cells on respiratory distress syndrome in newborn swines via the JAK-STAT signaling pathway. *European Review for Medical and Pharmacological Sciences*.

[95] Lopes-Pacheco M., Robba C., Rocco P. R. M., Pelosi P. (2020). Current understanding of the therapeutic benefits of mesenchymal stem cells in acute respiratory distress syndrome. *Cell Biology and Toxicology*.

[96] Khatri M., Richardson L. A., Meulia T. (2018). Mesenchymal stem cell-derived extracellular vesicles attenuate influenza virus-induced acute lung injury in a pig model. *Stem Cell Research & Therapy*.

[97] Loy H., Kuok D. I. T., Hui K. P. Y. (2019). Therapeutic implications of human umbilical cord mesenchymal stromal cells in attenuating influenza A(H5N1) virus-associated acute lung injury. *The Journal of Infectious Diseases*.

[98] Chan M. C., Kuok D. I., Leung C. Y. (2016). Human mesenchymal stromal cells reduce influenza A H5N1-associated acute lung injury in vitro and in vivo. *Proceedings of the National Academy of Sciences*.

[99] Can A., Coskun H. (2020). The rationale of using mesenchymal stem cells in patients with COVID-19-related acute respiratory distress syndrome: what to expect. *Stem Cells Translational Medicine*.

[100] Leng Z., Zhu R., Hou W. (2020). Transplantation of ACE2(-) mesenchymal stem cells improves the outcome of patients with COVID-19 pneumonia. *Aging and Disease*.

[101] Meng F., Xu R., Wang S. (2020). Human umbilical cord-derived mesenchymal stem cell therapy in patients with COVID-19: a phase 1 clinical trial. *Signal Transduction and Targeted Therapy*.

[102] Wang W., Lei W., Jiang L. (2021). Therapeutic mechanisms of mesenchymal stem cells in acute respiratory distress syndrome reveal potentials for Covid-19 treatment. *Journal of Translational Medicine*.

[103] Chen S., Fang L., Guo W. (2018). Control of T_reg_ cell homeostasis and immune equilibrium by Lkb1 in dendritic cells. *Nature Communications*.

[104] Zhang Q., Shi S., Liu Y. (2009). Mesenchymal stem cells derived from human gingiva are capable of immunomodulatory functions and ameliorate inflammation-related tissue destruction in experimental colitis. *The Journal of Immunology*.

[105] Zhao J., Chen J., Huang F. (2019). Human gingiva tissue-derived MSC ameliorates immune-mediated bone marrow failure of aplastic anemia *via* suppression of Th1 and Th17 cells and enhancement of CD4+Foxp3+ regulatory T cells differentiation. *American Journal of Translational Research*.

[106] Luo Y., Wu W., Gu J. (2019). Human gingival tissue-derived MSC suppress osteoclastogenesis and bone erosion *via* CD39-adenosine signal pathway in autoimmune arthritis. *EBioMedicine*.

[107] Zhang W., Zhou L., Dang J. (2017). Human Gingiva-Derived Mesenchymal Stem Cells Ameliorate Streptozoticin- induced T1DM in mice *via* Suppression of T effector cells and Up-regulating Treg Subsets. *Scientific Reports*.

[108] Su W., Li Z., Jia Y. (2017). MicroRNA-21a-5p/PDCD4 axis regulates mesenchymal stem cell-induced neuroprotection in acute glaucoma. *Journal of Molecular Cell Biology*.

[109] Lu Y., Xu Y., Zhang S. (2019). Human gingiva-derived mesenchymal stem cells alleviate inflammatory bowel disease via IL-10 signalling-dependent modulation of immune cells. *Scandinavian Journal of Immunology*.

[110] Bao Q., Hu P., Xu Y. (2018). Simultaneous blood-brain barrier crossing and protection for stroke treatment based on edaravone-loaded ceria nanoparticles. *ACS Nano*.

[111] Tomar G. B., Srivastava R. K., Gupta N. (2010). Human gingiva-derived mesenchymal stem cells are superior to bone marrow- derived mesenchymal stem cells for cell therapy in regenerative medicine. *Biochemical and Biophysical Research Communications*.

[112] Zhao J., Wang J., Dang J. (2019). A preclinical study-systemic evaluation of safety on mesenchymal stem cells derived from human gingiva tissue. *Stem Cell Research & Therapy*.

